# PlastiCRISPR: Genome Editing-Based Plastic Waste Management with Implications in Polyethylene Terephthalate (PET) Degradation

**DOI:** 10.3390/biom15050684

**Published:** 2025-05-08

**Authors:** Puja Palit, Maya Minkara, Maisha Abida, Safa Marwa, Chandrima Sen, Ayan Roy, Md Ridoan Pasha, Paulraj Selvakumar Mosae, Ayan Saha, Jannatul Ferdoush

**Affiliations:** 1 Department of Biological Sciences, Asian University for Women, Chittagong 4000, Bangladesh; puja.palit@auw.edu.bd (P.P.); maisha.abida@auw.edu.bd (M.A.); safa.marwa2022@auw.edu.bd (S.M.); chandrima.sen@auw.edu.bd (C.S.); ayanroy.bio@gmail.com (A.R.); 2 Department of Biology, Geology, and Environmental Science, University of Tennessee at Chattanooga, 615 McCallie Ave, Chattanooga, TN 37403, USA; tbq364@mocs.utc.edu; 3 Department of Physiology, Biochemistry, and Pharmacology, Chattogram Veterinary and Animal Sciences University, Khulshi, Chattogram 4225, Bangladesh; pasha.dpbp@cvasu.ac.bd; 4 Department of Environmental Sciences, Asian University for Women, Chittagong 4000, Bangladesh; p.selvakumar@auw.edu.bd

**Keywords:** PlastiCRISPR, genome editing, CRISPR-Cas9, microorganisms, biodegradation, bioplastic production, environmental sustainability, biotechnology

## Abstract

Plastic pollution has become a significant environmental issue worldwide, with global plastic production expected to reach 1800 million tons by 2050. One of the most commonly used plastics in the world is polyethylene terephthalate (PET), a synthetic polymer that is extremely durable but difficult to degrade. Thus, PET is dangerous to human health. To address this crisis, innovative approaches are being developed, including genome editing technologies. One of the recently advanced genome editing technologies is PlastiCRISPR, a novel concept that applies CRISPR-based genome editing to transform plastic waste management. PlastiCRISPR utilizes microorganisms to degrade plastic, generating valuable bioproducts like biofuels and biochemicals. Thus, this technology offers a sustainable solution because of its simple design, adequacy, and low cost, which can be integrated into existing waste management systems. Importantly, this review focuses on the PlastiCRISPR-based management of PET because it could drastically lower plastic waste, sustain natural resources by decreasing the requirement for plastic production, minimize energy intake, etc. Overall, this review provides an overview of the principles, applications, challenges, and future prospects of PlastiCRISPR in combating plastic pollution and shaping a more sustainable future.

## 1. Introduction

Plastic pollution has emerged as one of the most significant environmental issues of of the modern era, with its widespread presence in oceans, landfills, and ecosystems around the world. Despite efforts to reduce its impact through recycling and waste management, the enormous amount of plastic waste continues to grow, posing a substantial threat to global sustainability. By 2050, the world’s plastic production is expected to reach 1800 million tons, resulting in 12,000 million metric tons of plastic contaminating the environment. Additionally, nearly 6.4 million tons of waste enter the ocean every year [1]. One of the most commonly used plastics in the world is polyethylene terephthalate (PET), a synthetic polymer that is extremely durable but difficult to degrade. Thus, PET is dangerous to human health. In response to this urgent need, innovative approaches to reducing plastic pollution are being developed from various perspectives. Among these innovative approaches, genome editing technologies have received increased attention due to their potential to tackle environmental challenges. One such groundbreaking approach is PlastiCRISPR, a novel concept that utilizes CRISPR-based genome editing to transform how we manage plastic waste.

The concept of PlastiCRISPR is built upon the principles of a circular economy where waste is minimized and resources are conserved. By using microorganisms to degrade plastic, PlastiCRISPR offers a sustainable solution that can be integrated into existing waste management systems. This approach not only reduces plastic waste but also generates valuable bioproducts, such as biofuels and biochemicals, from broken-down plastic polymers. The PlastiCRISPR-Cas9 system is a fast, simple, and effective gene-editing approach. The Cas9 protein, derived from the type II CRISPR bacterial immune system, is a powerful tool for genome construction in other organisms. Cas9, an RNA-guided DNA incision enzyme, may be programmed to locate new loci [1]. Its use as a tool facilitates gene editing based on specific sequences. Nuclease-inactivated forms of Cas9 offer a versatile RNA-guided DNA-targeting platform for regulating, visualizing, and rewriting epigenetic states in a sequence-specific manner [1]. PlastiCRISPR provides a pathway to a more sustainable and resilient future by harnessing microorganisms’ intrinsic capabilities and enhancing them through targeted genetic alterations. With its potential to revolutionize plastic waste management and detoxify environmental pollutants, PlastiCRISPR heralds a new era of circularity and ecological sustainability. The management of polyethylene terephthalate (PET) is the primary focus of this research since it has the potential to significantly reduce plastic waste, preserve natural resources by reducing the need for plastic manufacture, minimize energy use.

This review provides background information on the principles, applications, challenges, and future prospects of PlastiCRISPR as a transformative solution for advancing circular economy approaches to plastic waste management. By examining the latest research developments and insights from the intersection of genome editing and sustainability, we aim to deliver a comprehensive overview of the PlastiCRISPR’s potential in combating the global plastic waste crisis and shaping a more sustainable future for generations to come.

## 2. Genome Editing in Plastic Waste Management

Genome editing, a powerful tool in biotechnology, has the potential to revolutionize plastic waste management by enabling the development of microorganisms capable of breaking down plastics into harmless components. This technology has gained popularity in recent years, particularly in relation to addressing the severe issue of plastic pollution in marine habitats. Researchers are employing gene editing methods to create microbes that can degrade plastics more efficiently and sustainably. This approach involves manipulating organisms to express specific genes associated with microplastic breakdown, such as polyethylene terephthalate hydrolase, dehalogenase, esterase, depolymerase, and laccase [2]. In light of the pressing necessity for sustainable solutions to plastic pollution, utilizing genome editing to improve microbial plastic degradation seems to be a highly promising and unique avenue for future environmental biotechnology.

One of the key organisms being engineered for plastic degradation is the bacterium *Ideonella sakaiensis*, which has been found to break down PET into its constituent monomers [3]. The French (Biopôle Clermont-Limagne) company Carbios has developed a process based on an enzyme called LCC (leaf-branch compost cutinase), identified in 2012 by Japanese researchers, which is particularly effective at degrading PET [3]. The company has also partnered with L’Oréal (Clichy, France) and Nestlé (Vevey, Switzerland) to produce enzymatically recycled food-grade PET plastic, demonstrating the potential of this technology [4]. Gene editing tools such as CRISPR-Cas9 have made it easier to manipulate organisms, allowing for the incorporation of genes that encode enzymes involved in microplastic degradation. For instance, a strain of *Pseudomonas aeruginosa* bacteria was genetically engineered to increase the formation of sticky exopolymeric substances, enhancing its capacity to accumulate microplastics in its biofilm [2]. These advancements underscore that enzyme-based plastic recycling is not merely a theoretical notion but a swiftly progressing reality, providing insight into how industry partnerships might expedite the shift toward sustainable plastic waste management.

The use of genome editing in plastic waste management offers several advantages, including the potential for more efficient and specific degradation of plastics, reduced energy consumption, and the ability to target specific types of plastics. However, it also raises questions about the patentability of living organisms and the risks of releasing genetically modified microorganisms into the environment [3]. Overall, genome editing is a promising field of research that could help to establish long-term plastic waste management techniques. Scientists may be able to construct microorganisms that can digest plastics more effectively using gene editing, thereby minimizing the environmental impact of plastic pollution. The utilization of modern genome editing procedures such as CRISPR-Cas9 represents a significant advancement, facilitating the accurate and effective augmentation of microbial abilities for microplastic breakdown and creating new opportunities for environmental remediation.

## 3. PlastiCRISPR Technology

**Figure 1:**
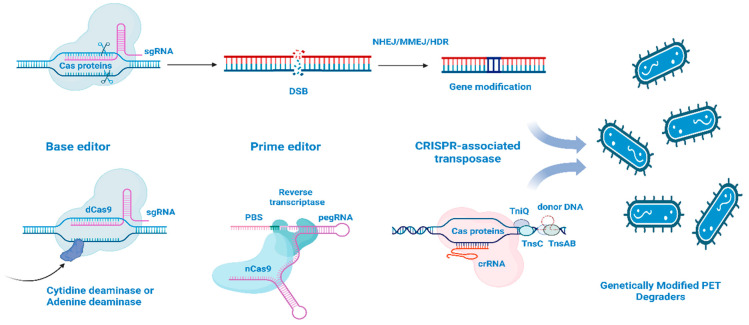
CRISPR-Cas9 system, a Tool for microbial genetic engineering. This figure depicts the CRISPR-Cas9 gene editing mechanism and its applications in microbial engineering. It illustrates the key components, including the Cas9 protein and guide RNA (gRNA), which directs Cas9 to specific DNA sequences in a microorganism’s genome. Upon binding to the target DNA, Cas9 creates a double-strand break (DSB), which the cell repairs through either non-homologous end joining (NHEJ) or homology-directed repair (HDR). NHEJ can lead to gene knockouts, while HDR enables precise modifications when a repair template is provided.

The mechanism of PlastiCRISPR in plastic waste management involves introducing specific guide RNA (gRNA) sequences that target and bind to the desired genetic sequences within the microorganisms. The Cas9 enzyme then cuts the targeted DNA, allowing for the insertion or deletion of genetic material, which can be used to enhance the microorganisms’ ability to produce enzymes capable of breaking down plastic polymers [5]. This approach has been explored in the degradation of PET, polystyrene, and other recalcitrant plastic materials [6]. Researchers have previously modified bacteria and fungi using CRISPR-based techniques to enhance their plastic-degrading capabilities. For instance, as shown in Figure 1, researchers have used CRISPR to engineer *Escherichia coli* and *Pseudomonas putida* strains to express PET-degrading enzymes, leading to the enhanced biodegradation of PET [2]. A CRISPR-based transposon technology (VcTn6677) from *Vibrio cholerae*, for example, allowed for the site-specific integration of gene cassettes carrying the Lpp promoter and signal peptide upstream of PET-hydrolyzing genes into the *E. coli* genome. This technique ensured stable, surface-displayed, continuous enzyme synthesis without the need for inducers or antibiotics [6].

Similarly, CRISPR has been employed to enhance the production of polyhydroxyalkanoates (PHAs), a class of bioplastics, by modifying the metabolic pathways of microorganisms. For example, using the CRISPR/Cas9n-λ-Red genome editing strategy (CRP) approach, the KTc9n20 strain of *Pseudomonas putida* KT2440 was developed by regulating the expression of nine distinct genes, divided into four engineered modules, to improve ferulic acid-to-PHA conversion. To achieve high-efficiency and scarless genome editing in *Pseudomonas putida* KT2440, the *pyrF* selection marker was used. Cas9n was employed to reduce lethality and off-target effects. Cas9n with λ-Red recombination proteins were integrated into the genome to enhance plasmid stability. Furthermore, the CRP technique enabled quick plasmid curing in conjunction with genome editing, thereby significantly simplifying and acceleratingd accelerated the overall procedure [7].

The potential of CRISPR to revolutionize environmental science extends beyond plastic waste management, encompassing applications in biofuels, bioplastics, bioremediation, and other environmental challenges. PlastiCRISPR has also been utilized to engineer microbes capable of converting plastic waste into valuable chemicals or biofuels [8]. By targeting specific metabolic pathways involved in aromatic compound degradation and biosynthetic processes, researchers have created strains of *Escherichia coli* and *Pseudomonas putida* that can transform terephthalic acid, a PET degradation product, into useful compounds like muconic acid and PHA. Intermediates like muconic acid are produced that can be used in polymer synthesis. Some intermediates are further converted into acetyl-CoA, which feeds into the PHA biosynthetic pathway to produce biodegradable plastics.

Research has also shown that plasticizers, which are additives used to increase the flexibility, softness, and workability of materials, primarily plastics, are widely present in the environment and can hinder the recycling of plastics. For example, some plasticizers, particularly phthalates, have been linked to health risks like endocrine disruption [9]. Their presence can complicate the recycling process due to the need for separation and purification steps. The hazardous properties of plasticizers highlight the importance of developing advanced technologies like PlastiCRISPR to mitigate the environmental impact of plastic waste and improve recycling efforts [10]. Thus, PlastiCRISPR, a recently advanced genome editing technology, has been utilized in managing plastic waste, including PET. While PlastiCRISPR shows great potential in improving microbial capacities for plastic breakdown and valorization, various difficulties must be overcome before it can be widely used. These difficulties include ensuring the genetic stability of modified strains under changing environmental conditions, addressing biosafety and regulatory issues associated with the release of genetically modified organisms, and overcoming technical challenges such as the effective breakdown of complex plastic additives like plasticizers.

## 4. Molecular Mechanisms of Genome Editing for Enhanced Plastic Degradation

**Figure 2:**
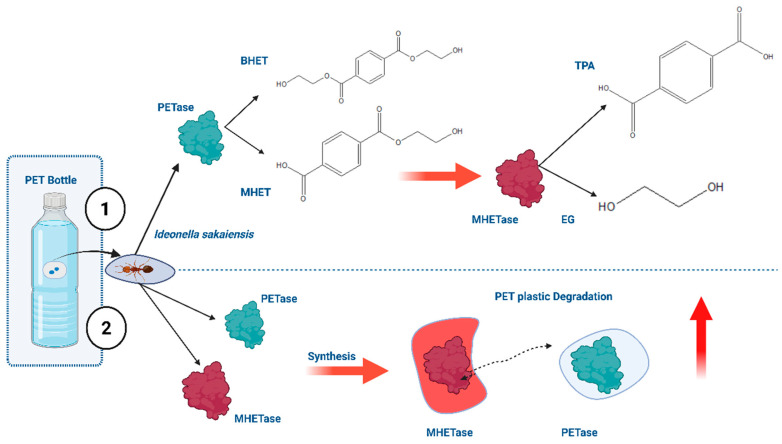
Enzymatic degradation pathway of polyethylene terephthalate (PET). The diagram illustrates the enzymatic degradation of polyethylene terephthalate (PET) by *Ideonella sakaiensis*. Initially, the enzyme PETase hydrolyzes PET into bis-(2-hydroxyethyl) terephthalate (BHET) and mono-(2-hydroxyethyl) terephthalic acid (MHET). This is followed by the action of MHETase, which further breaks down MHET into terephthalic acid (TPA) and ethylene glycol. These enzymes together represent a natural plastic degradation pathway and demonstrate their potential for industrial recycling, allowing PET waste to be recycled back into high-quality monomeric building blocks.

The degradation of durable plastic polymers such as polyethylene terephthalate (PET), polyethylene (PE), and polystyrene (PS) remains a critical environmental challenge due to their chemical stability, hydrophobic surfaces, and resistance to microbial attack [11]. Conventional microbial metabolism is often inadequate for efficiently degrading such polymers. However, several bacterial strains, such as *Ideonella sakaiensis*, *Bacillus pumilus*, *Pseudomonas putida*, and *Escherichia coli*, have shown promising plastic-degrading capabilities via enzyme-mediated hydrolysis. Specifically, enzymes like PETase and MHETase, identified in *I. sakaiensis*, catalyze the breakdown of PET into its monomeric forms—terephthalic acid (TPA) and ethylene glycol (EG)—enabling its biological recycling [12,13]. PETase first hydrolyzes PET into bis-(2-hydroxyethyl) terephthalate (BHET) and mono-(2-hydroxyethyl) terephthalate (MHET), which MHETase then converts into TPA and EG (Figure 2). These degradation steps are central to the enzymatic recycling process and serve as a model for engineering robust microbial systems.

Advancements in CRISPR-Cas9 genome editing have accelerated the functional enhancement of these plastic-degrading microorganisms. CRISPR-Cas9 enables targeted DNA modifications by introducing double-stranded breaks at specified genomic loci, which are subsequently repaired via non-homologous end joining (NHEJ) or homology-directed repair (HDR) mechanisms [14,15]. In *H. bluephagenesis* TD01, a refined CRISPR-Cas9-NHEJ editing system reached a high efficiency of 31.3% and enabling the rapid and effective deletion of DNA fragments up to 50 kb. Three sgRNAs that target the Crick and Watson strands were used in conjunction with the background activity of the homologous recombination (HR) repair mechanism and the Cas9 expression that is triggered by the arabinose-inducible Para promoter to achieve this [16]. This allows scientists to precisely insert or knock out genes involved in plastic degradation. For instance, researchers have engineered *P. putida* to enhance its metabolic capacity for TPA assimilation by deleting competing catabolic pathways and overexpressing PHA synthesis genes, thus converting waste PET into value-added bioplastics [17,18]. Similarly, in *E. coli*, CRISPR has been used to create fusion enzymes that combine PETase with MHETase or laccases, improving substrate affinity, thermal stability, and degradation rates under industrial conditions. For example, a whole-cell biocatalyst was developed by displaying a PETase mutant (FAST-PETase) and a carbohydrate-binding module 3 (CBM3) on the surface of *E. coli* cells, combined with the intracellular expression of MHETase, to create a bacterial enzyme cascade reaction system (BECRS) for PET degradation [19]. These engineered strains can degrade PET more rapidly, even under stress conditions such as elevated temperatures or varying pH levels.

Moreover, CRISPR offers the ability to manipulate entire metabolic networks and regulatory systems. Through promoter engineering, ribosome binding site (RBS) tuning, and gene circuit design, the expression of plastic-degrading genes can be made inducible, constitutive, or environmentally responsive [20]. Synthetic biology approaches have integrated CRISPR into programmable logic gates, controlling enzyme expression in response to plastic-derived cues—allowing for resource-efficient degradation only when plastic substrates are detected [21]. CRISPR interference (CRISPRi) in *Pseudomonas putida* downregulates competing pathways (e.g., β-oxidation) that reroute carbon flux to polyhydroxyalkanoate (PHA) production from plastic monomers. These pathway optimizations serve as examples of the iterative design–build–test–learn cycle of synthetic biology to maximize plastic-to-product conversion rates [22]. CRISPRi also allows for the precise manipulation of genes involved in PHA production, providing control over polymer composition and attributes such as by changing the 3-hydroxybutyrate (3HB)/4-hydroxybutyrate (4HB) ratio or polyhydroxybutyrate (PHB) molecular weight. This fine-tuning improves the development of biodegradable plastics for a range of industrial applications [23]. Moreover, by competing for critical cellular resources, the CRISPR-driven expression of plastic-degrading enzymes such as PETase and cytochrome P450 leads to increased metabolic loads by reducing growth rates and bioproduct yields [24,25]. Enzyme-constrained genome-scale metabolic models (ecGEMs), dynamic control systems used to maximize resource allocation and pathway flux, and enzyme expression modifications are some of the ways synthetic biology addresses these issues. Examples include the use of quorum-sensing-regulated promoters to postpone enzyme production until after peak biomass accumulation and the identification of lysine biosynthesis bottlenecks in *Saccharomyces cerevisiae* using ecGEMs [26]. Additionally, genome editing can enhance traits such as membrane transport, substrate uptake, and the efflux of toxic byproducts, enabling improved survival and performance of genetically modified strains in real environmental or industrial settings.

Another frontier in this area involves adaptive genome editing for stress resistance and cross-pathway integration. For example, CRISPR has been used to upregulate genes responsible for oxidative stress resistance or to engineer quorum-sensing pathways, enabling genetically edited bacteria to survive better in harsh or fluctuating environments, such as marine ecosystems or landfill sites [27]. Genome editing can also optimize enzyme secretion systems (such as TAT or Sec pathways) to increase extracellular enzyme concentrations, allowing for the effective degradation of insoluble plastics in surrounding environments. In bioreactor setups, CRISPR-engineered microbial consortia have demonstrated cooperative plastic degradation, where one strain hydrolyzes plastic, and another assimilates the resulting monomers into biofuels, bioplastics, or other biomaterials [28]. In addition to enzyme pathway engineering, researchers are now applying CRISPR for the directed evolution of plastic-degrading proteins. This involves iteratively mutating and screening gene variants to select those with enhanced catalytic properties, pH tolerance, or thermal resilience [29,30]. Structural modeling and AI-guided design tools now aid in predicting optimal mutation sites, which are then implemented through genome editing to generate hyperstable or multifunctional enzymes. Although long-term stability, environmental safety, and scalability are critical for real-world applications, adaptive genome editing, and AI-driven enzyme engineering show great promise.

Overall, genome editing—particularly through CRISPR-Cas technologies—has revolutionized the development of microbial platforms for plastic degradation. From targeted gene integration and synthetic gene circuits to stress-adaptive genome editing and enzyme optimization, this approach provides a powerful toolkit for redesigning microorganisms into efficient agents of bioremediation. When integrated with the principles of the circular economy and industrial bioprocessing, these technologies could unlock a sustainable future in which persistent plastic waste is upcycled into valuable bioproducts—thereby reducing the ecological burden and advancing environmental biotechnology.

## 5. PlastiCRISPR and Artificial Intelligence: Plastic Waste Management in the Circular Economy

Circular economy frameworks are increasingly recognized as essential strategies for addressing escalating environmental concerns and resource depletion. Based on the principles of reduce, reuse, and recycle, the circular economy emphasizes closed-loop systems that prioritize sustainable inputs, regenerative cycles, and minimized waste outputs [31]. Within this model, plastic waste management remains one of the most pressing global challenges.

PlastiCRISPR can significantly increase the rate and efficiency of PET breakdown, offering a sustainable alternative to traditional chemical recycling [32]. The potential of PlastiCRISPR extends beyond single-enzyme enhancement. By engineering synthetic microbial consortia, researchers can create synergistic biodegradation systems that mimic natural microbial communities, improving substrate utilization and metabolic efficiency [33]. These biological innovations can be incorporated into industrial-scale waste treatment processes, aligning seamlessly with circular economy goals [34].

Artificial intelligence (AI) plays a pivotal role in accelerating and optimizing the PlastiCRISPR pipeline. AI-powered computational platforms can predict the effects of specific gene edits on enzyme function and stability using molecular dynamics simulations and structure–function modeling [35]. Machine learning algorithms can analyze high-throughput screening data to identify the most effective gene modifications, saving significant time and experimental resources [36]. Moreover, AI enables the design of smart bioreactors and adaptive process controls in plastic degradation facilities. With real-time data from sensors and IoT devices, AI systems can monitor microbial activity, substrate concentration, pH, temperature, and by-product levels, dynamically adjusting operational conditions for optimal enzyme performance and degradation efficiency.

Beyond the lab, AI facilitates the automation of plastic waste identification and sorting through computer vision and robotics. These technologies enhance the efficiency of material recovery facilities (MRFs) by accurately classifying plastic types and separating materials suitable for bio-degradation versus those for mechanical recycling [37]. As more data are processed, AI systems keep learning and developing, enabling them to adapt to new forms of plastics and changing waste sources. This approach ensures maximum material utilization and aligns with the life-cycle thinking central to circular economy models.

The integration of PlastiCRISPR and AI also supports life-cycle assessment (LCA) and techno-economic analysis (TEA), offering insights into environmental impact, cost-effectiveness, and scalability. These assessments help identify the most promising microbial strains and editing strategies, ensuring that only sustainable and economically viable solutions progress to industrial application [38].

In summary, PlastiCRISPR, enhanced by AI technologies, represents a transformative advancement in circular waste management. By combining synthetic biology, machine learning, and process automation, this approach holds the potential to revolutionize how societies manage plastic waste—minimizing landfill dependence, reducing microplastic pollution, and closing the loop on synthetic polymer use. Understanding the molecular mechanisms of microbial plastic degradation, in conjunction with AI-optimized system design, will be essential for developing the next generation of sustainable biotechnological solutions.

## 6. Potential Applications and Scalability

The PETase gene holds significant promise for advancing circular solutions in plastic waste management. Research by Orlando et al. highlights the microbial enzyme biotechnology’s potential to achieve plastic waste circularity through the utilization of PETase. This enzyme, with its ability to catalyze the breakdown of PET into its monomers, offers a sustainable approach to recycling plastic waste [17]. The versatility of PETase extends to its effectiveness in degrading PET in saltwater conditions, making it a valuable tool for addressing plastic pollution in marine environments. The future of PETase gene editing holds promise for further enhancing its efficiency and scalability through CRISPR technology. By focusing on the potential applications and scalability of the PETase gene, PlastiCRISPR could revolutionize the circular economy in plastic waste management.

The engineering of microorganisms to enhance their ability to degrade plastics has led to significant advancements in plastic waste management. Notable organisms, such as *Ideonella sakaiensis* and *Pseudomonas putida*, have been modified to improve their efficiency in breaking down various plastic substrates. Table 1 highlights some of these engineered microorganisms and their products, pathways, substrates, and relevant references for further exploration.

## 7. Environmental Benefits of Using PlastiCRISPR for Enhanced Plastic Degradation

**Figure 3:**
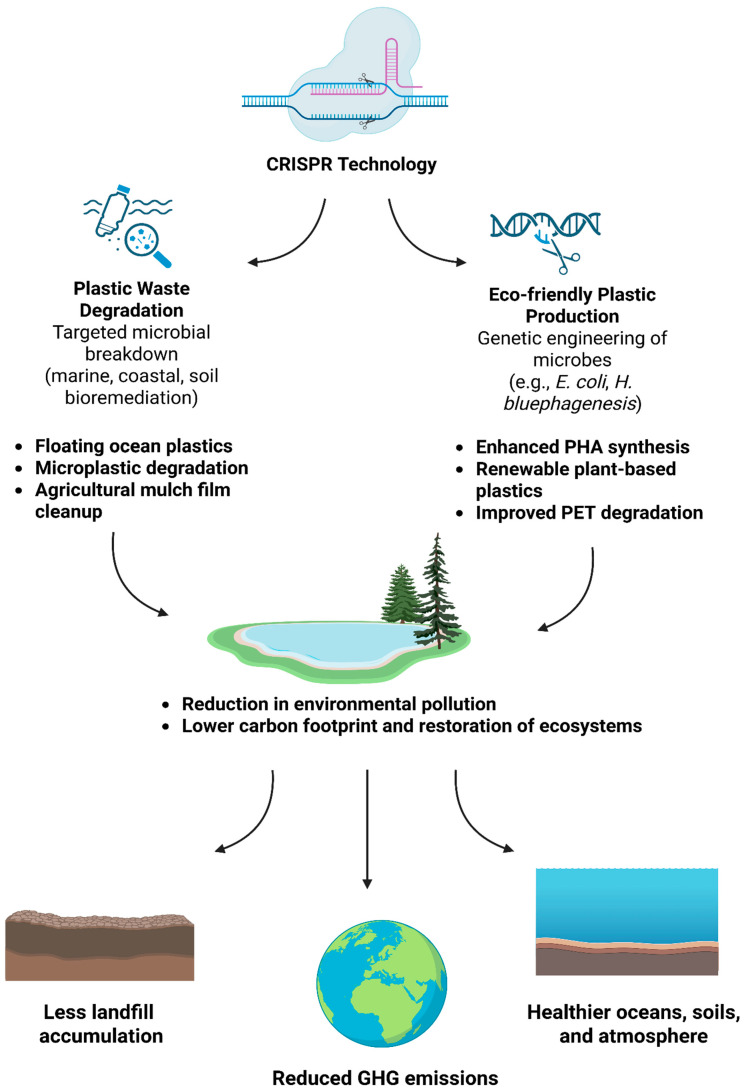
Role of CRISPR technology in advancing plastic waste management and sustainable plastic production. CRISPR-based genetic engineering enables two major strategies for addressing plastic pollution: (1) plastic waste degradation through targeted microbial breakdown of marine, coastal, and soil plastics, including floating ocean plastics, microplastics, and agricultural mulch films, and (2) eco-friendly plastic production by enhancing the microbial synthesis of biodegradable plastics such as polyhydroxyalkanoates (PHAs), creating renewable plant-based plastics, and improving the degradation efficiency of polyethylene terephthalate (PET). These approaches collectively contribute to environmental benefits, including reduced landfill accumulation, lower greenhouse gas (GHG) emissions, and healthier ecosystems.

The use of plastics in daily life is convenient, but it also greatly pollutes the environment when a large amount of plastic waste is produced [48]. Conventional plastics are made from polymers that degrade extremely slowly, remaining in the environment for many years. Reports indicate that between 4.8 and 12.8 million metric tons of plastic waste are dumped into the ocean each year, often without effective management plans. This disposal of plastic waste has various detrimental effects on marine organisms [2]. The specific method of using CRISPR to degrade plastic shows enormous potential for cleaning up oceans and streams. CRISPR technology efficiently reduces the ecological burden of plastic pollution by focusing on plastic waste that is floating on the surface of the water or accumulating along the coasts (Figure 3). CRISPR-designed targeted microbial consortia may also be added to contaminated agricultural soils, where they can break down microplastics and leftover plastic mulch films, gradually enhancing soil health and agricultural output. For example, CRISPR-Cas9 technology can genetically modify *Phanerochaete chrysosporium* by increasing the expression of its laccase and peroxidase genes—two essential enzymes involved in lignin breakdown. The resulting modified strain has shown significant advancements in biotreating lignin-rich industrial waste by efficiently breaking down lignin into simpler components [27]. While conventional petroleum-derived plastics typically lack lignin, modern bio-composite plastics increasingly incorporate lignin to enhance sustainability and biodegradability.

CRISPR technology not only reduces plastic pollution but also promotes the production of eco-friendly plastics made from renewable resources, which lowers carbon emissions. The CRISPR-Cas9 system, a powerful tool for genome editing, can efficiently insert or delete genes to enhance carbon flow for PHA synthesis. *E. coli* has been modified by overexpressing *pntAB* and deleting four byproduct-related genes (*pflB*, *ldhA*, *adhE*, and *fnr*) in order to improve PHA production and facilitate substrate conversion. Multiple route alterations have proven efficient, as the resultant strain, HR002, has shown enhanced cell growth and PHA content, produced fewer byproducts, and accumulated more acetyl-CoA [49]. CRISPR-edited enzymes also improve thermal stability and activity, accelerating the breakdown of polyethylene terephthalate (PET) into reusable monomers [50]. Researchers have achieved a 100% effective CRISPR-Cas9-based genome editing technique, demonstrating remarkable efficacy in increasing the necessary monomer content in *H. bluephagenesis*-derived PHA copolymers [33]. Furthermore, CRISPR-modified microbial strains (such as X-32) break down polyolefins, polyesters, and polyamides into harmless biomass, water, and carbon dioxide. Unlike polymers with cleavable links, inert plastics (polyolefins, polyesters, and polyamides) are the most difficult to upcycle since they cannot be easily broken down chemically or enzymatically. As a result, they are usually disposed of in landfills or burned, resulting in significant greenhouse gas emissions [51].

Hence, by leveraging CRISPR technology to enhance the production of eco-friendly plastics from renewable resources like plant-based components, we can reduce our dependence on fossil fuels and minimize the carbon footprint of plastic production.

## 8. PlastiCRISPR-Based Bioplastic Production and Its Comparison with Traditional Methods

Bioplastics, in contrast to synthetic plastics made from petroleum and fossil fuels, are polymers derived from renewable natural sources like plant cellulose and bacterial byproducts. Some bioplastics are biocompatible, meaning they are non-toxic to living tissues, while others are biodegradable and can be composted [52]. Most bioplastic issues may be resolved using CRISPR-Cas9 (RNA-guided endonucleases) by modifying the genes that encode the enzymes that produce compounds of interest in biopolymers, such as polyhydroxyalkanoates [53]. By applying CRISPR-Cas9 to specific microbes or plants, researchers may enhance the production of bioplastic materials, achieving outcomes such as increased bioplastic accumulation in *E. coli* or improved biopolymer yield from plant biomass. For example, researchers have adapted CRISPR-Cas9 in the oil-producing yeast *Yarrowia lipolytica* to improve the modification of genes, facilitating the efficient generation of lipids and hydrocarbons—essential precursors for bioplastics and specialty polymers [54]. In another study, CRISPR-Cas9 was utilized to knock out the *gbss* gene in potatoes, producing amylose-free starch with a smoother texture. Such customized starch qualities enhance applicability for industrial biopolymer applications, indirectly increasing bioplastic production from plant biomass [55]. Thus, to improve the quality, yield, or efficiency of bioplastic manufacturing, certain genes connected with biopolymer production pathways can be changed through targeted genome editing.

This enables the development of bioplastics with enhanced resilience, malleability, or biodegradability that are suited for particular applications. In brief, CRISPR gene editing offers a simplified and enhanced approach to bioplastic production, promoting the development of sustainable and efficient alternatives to conventional plastics through its precise modification of genetic material.

Traditional methods for managing plastic waste, such as landfill disposal, recycling, and incineration, have limitations in terms of efficiency, sustainability, and environmental impact [36]. PlastiCRISPR, on the other hand, offers a promising alternative by targeting the genetic makeup of plastic-degrading microorganisms, thereby enhancing their ability to break down plastics efficiently and effectively [56]. PlastiCRISPR harnesses the power of genetic engineering to customize microorganisms for specific plastic degradation pathways, which increases the speed and efficacy of plastic degradation and reduces the overall environmental footprint associated with traditional plastic waste management practices [57]. This novel approach not only addresses the important issue of plastic pollution but also provides a path for more sustainable waste management options, as seen in Table 2 by comparing PlastiCRISPR to traditional methods.

## 9. Ethical Implications

PlastiCRISPR raises several important ethical considerations, particularly related to environmental safety, organism welfare, and equitable access. The environmental release of genetically modified microorganisms poses potential risks to ecosystems, underscoring the need for comprehensive risk assessments and regulatory oversight. Moreover, the welfare of these engineered organisms must be carefully monitored to avoid unintended biological harm. Equitable access to PlastiCRISPR technology is also crucial—especially for communities disproportionately impacted by plastic pollution—ensuring they have a voice in policy development and deployment strategiess [39]. Ultimately, the responsible application of PlastiCRISPR depends on addressing these ethical challenges thoughtfully and transparently to maximize its environmental benefits while minimizing potential risks [58].

## 10. Conclusions and Future Direction

PlastiCRISPR research is evolving toward optimizing microbial biotechnologies for plastic degradation and valorization. It also includes developing alternative plastics from non-food biomass and utilizing genome editing for plastic recycling, aiming to combat plastic pollution sustainably. Comprehensive environmental risk assessments, interdisciplinary collaboration, and further research and development are necessary to ensure the safe and effective deployment of genome-edited microorganisms for plastic degradation, ultimately leading to a more sustainable future in combating the global plastic waste crisis. Genome editing technologies such as CRISPR-Cas9 can be utilized to modify the genes of microorganisms to enhance their ability to degrade plastics, leading to the development of more efficient and cost-effective recycling processes. However, there are still challenges to be addressed in the development of genome editing-based plastic waste management solutions. For instance, the deconstruction of plastics using microorganisms can release additives that must be managed separately [12]. Additionally, the costs of these approaches are currently higher than production from fossil resources, and incentives will be necessary to encourage manufacturers to adopt these methods. In the context of circular solutions for plastic waste, genome editing technologies can be used to modify the genes of microorganisms to enhance their ability to degrade plastics, leading to the development of more efficient and cost-effective bio-recycling processes.

**Figure 4:**
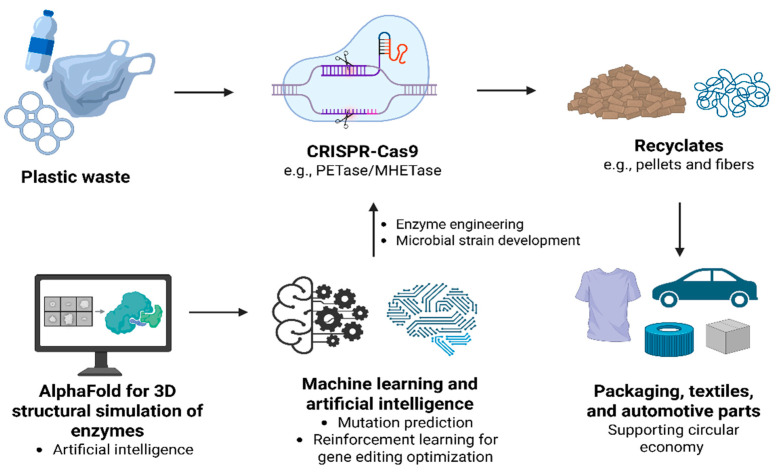
Synergistic application of PlastiCRISPR and artificial intelligence for sustainable plastic recycling. Plastic waste is enzymatically degraded using CRISPR-Cas9-engineered enzymes such as PETase/MHETase, resulting in high-quality recyclates (e.g., pellets and fibers). These recyclates are repurposed into manufacturing sectors such as packaging, textiles, and automotive parts, supporting a circular economy. Artificial intelligence tools, including AlphaFold2 for 3D structural simulations, machine learning for mutation prediction, and reinforcement learning for gene editing optimization, accelerate enzyme engineering and microbial strain development, enhancing plastic degradation efficiency.

In the future, innovations in PlastiCRISPR could focus on producing recyclates—processed recycled plastics in forms like pellets or fibers—with enhanced quality and usability (Figure 4). This would enable their use in diverse manufacturing applications, including packaging, textiles, and automotive parts, reducing dependence on virgin plastics and supporting corporate sustainability goals. AI technologies can also enhance PlastiCRISPR approaches through optimized microbial engineering, precision waste sorting, and ecosystem monitoring, creating synergistic solutions for plastic waste management. Machine learning (ML) and AI models predict optimal mutations in PETase/MHETase enzymes for CRISPR editing, increasing PET degradation efficiency compared to random mutagenesis approaches [19]. Tools like AlphaFold with ML can enable 3D structural simulations to guide CRISPR-Cas9 edits for improved thermal stability (up to a 55 °C operational range) [59]. Apart from that, reinforcement learning algorithms can identify optimal gene knock-in/knockout combinations in plastic-degrading microbes, reducing development time for novel strains [60]. The fusion of AI and PlastiCRISPR technologies can open a new era of smart, efficient, and sustainable plastic waste management, offering scalable solutions to the global plastic pollution crisis while advancing the circular economy.

In conclusion, PlastiCRISPR presents a transformative solution to plastic pollution by harnessing genome editing to enhance microbial plastic degradation and recycling capabilities. When integrated with AI, this approach becomes even more powerful—enabling predictive modeling, enzyme optimization, and adaptive system control for improved efficiency. While technical and ethical challenges remain, ongoing research continues to advance the scalability and safety of these innovations. Together, PlastiCRISPR and AI pave the way for an efficient, scalable, and sustainable plastic waste management strategy aligned with circular economy principles.

## Figures and Tables

**Table 1:** Engineered microorganisms for enhanced plastic degradation.

Organism	Product	Pathway	Substrate	Reference
*Streptomyces* spp.	Polyhydroxyalkanoates (PHA)	Biodegradation of PET	Pre-treated post-consumer PET	[39]
*Pseudomonas* spp.	Biopolymers	Hydrolysis and fermentation	Various plastic-related substrates	[3]
*Escherichia coli*	Degradation products	Enzymatic hydrolysis	Polyethylene, polystyrene	[13]
*Ideonella sakaiensis*	Monomers of PET	PET degradation	Polyethylene terephthalate (PET)	[12]
*Staphylococcus epidermidis* Un-C2-8	PET-degrading enzyme	Genetic engineering and expression	Polyethylene terephthalate (PET)	[40]
*Kineococcus endophyticus* Un-5	PET-degrading enzyme	Genetic engineering and expression	Polyethylene terephthalate (PET)	[40]
*Rhodococcus* spp.	Hydrolytic enzymes	Biodegradation	Various plastics	[41]
*Vibrio alginolyticus*	Hydrolytic enzymes	Biodegradation	Polyvinyl alcohol, LDPE	[42]
*Vibrio parahemolyticus*	Hydrolytic enzymes	Biodegradation	Polyvinyl alcohol, LDPE	[42]
*Bacillus pumilus*	Hydrolytic enzymes	Biodegradation	Low-density polyethylene (LDPE)	[43]
*Pseudomonas aeruginosa*	Hydrolytic enzymes	Biodegradation	Linear low-density polyethylene (LLDPE)	[44]
*Pseudomonas alloputida*	Hydrolytic enzymes	Biodegradation	Linear low-density polyethylene (LLDPE)	[44]
*Castellaniella denitrificans*	Enzymatic degradation products	Ligninolytic degradation	Linear low-density polyethylene (LLDPE)	[44]
*Debaryomyces hansenii*	Enzymatic degradation products	Ligninolytic degradation	Linear low-density polyethylene (LLDPE)	[44]
*Thermobifida fusca*	Enzymatic degradation products	Ligninolytic degradation	Polyethylene terephthalate (PET)	[45]
*Saccharomonospora viridis*	Hydrolytic enzymes	Biodegradation	Polyethylene terephthalate (PET)	[45]
*BIND-PETase*	TPA and MHET	Enzymatic hydrolysis	Polyethylene terephthalate (PET)	[46]
*Bacillus cereus*	Hydrolytic enzymes	Biodegradation	Low-density polyethylene (LDPE)	[47]

**Table 2:** Contrast analysis: PlastiCRISPR vs. traditional methods.

Aspect	PlastiCRISPR-Based Bioplastic Production and Degradation	Traditional Methods (Petrochemical Plastics and Waste Management)
Source Material	Renewable sources (e.g., plant biomass, bacteria)	Fossil fuels (petroleum, natural gas)
Technology Core	CRISPR-Cas9 genetic engineering of microbes and plants	Chemical polymerization for plastics; mechanical or thermal processes for recycling
Production Efficiency	Enhanced through gene editing (e.g., increased yield of PHAs in *E. coli*)	Limited by the physical and chemical processes; resource-intensive
Biodegradability	High: Engineered microbes improve degradation; bioplastics are compostable	Low: Most synthetic plastics persist for centuries in the environment
Environmental Impact	Reduced: Less carbon emission, less plastic pollution, targeted degradation	High: Pollution, microplastic contamination, greenhouse gas emissions
Customization Flexibility	High: Genetic edits allow tuning for resilience, malleability, and degradation speed	Low: Chemical properties fixed post-production
Waste Management Role	Proactive: Microbes genetically enhanced to degrade plastics	Reactive: Relies on incineration, landfill, or recycling
Scalability	Emerging: Lab-scale and pilot projects growing; scaling requires bioengineering infrastructure	Mature infrastructure but costly and energy-intensive
Economic Feasibility	Currently higher costs; potential for decrease with innovation and policy support	Economically efficient (currently) but with hidden environmental costs
Sustainability Alignment	Strong: Supports circular economy and green transitions	Weak: Linear economy model (produce–consume–dispose)
Public and Regulatory Acceptance	Developing: Concerns about GMOs and biosafety in open ecosystems	Well established but facing increasing regulation due to environmental harm

## Data Availability

Not applicable.
